# Knockdown of INPP5K compromises the differentiation of N2A cells

**DOI:** 10.3389/fnmol.2024.1356343

**Published:** 2024-03-15

**Authors:** Annamaria Manzolillo, Lennart Gresing, Christian A. Hübner, Patricia Franzka

**Affiliations:** ^1^Institute of Human Genetics, Jena University Hospital, Friedrich Schiller University, Jena, Germany; ^2^Center of Rare Diseases, Jena University Hospital, Friedrich Schiller University Jena, Jena, Germany

**Keywords:** INPP5K, brain, development, endoplasmic reticulum, glycosylation

## Abstract

Inositol polyphosphate 5-phosphatase K (INPP5K), also known as SKIP (skeletal muscle and kidney-enriched inositol phosphatase), is a cytoplasmic enzyme with 5-phosphatase activity toward phosphoinositides (PIs). Mutations in INPP5K are associated with autosomal recessive congenital muscular dystrophy with cataracts and intellectual disability (MDCCAID). Notably, muscular dystrophy is characterized by the hypoglycosylation of dystroglycan. Thus, far, the underlying mechanisms are only partially understood. In this study, we show that INPP5K expression increases during brain development. Knockdown of INPP5K in the neuroblastoma-derived cell line N2A impaired their neuronal-like differentiation and interfered with protein glycosylation.

## Introduction

Phosphoinositides (PIs) are signaling lipids derived from phosphatidylinositol, a ubiquitous phospholipid within the cytoplasmic leaflet of cellular membranes. Their intracellular levels are strictly regulated by specific PI kinases, phosphatases, and phospholipases. They act as integrators of membrane dynamics with a broad impact on all aspects of cell physiology, such as cytoskeleton organization, mitosis, transport processes, cell polarity, migration, and autophagy (Balla, 2013; Raghu et al., 2019; Posor et al., 2022). Recent discoveries indicate that dysfunctions in the control of their levels can result in different pathologies (Pendaries et al., 2003; Raghu et al., 2019). Mutations in INPP5K, the enzyme with 5-phosphatase activity toward PIs (Ijuin et al., 2000; Vandeput et al., 2006), were reported in patients suffering from congenital muscular dystrophy with cataracts and intellectual disability (MDCCAID) and short stature (Osborn et al., 2017; Wiessner et al., 2017). The morpholino knockdown in zebrafish embryos resulted in curled and shortened tails, impaired swimming and touch-evoked escape responses, smaller eyes, and altered skeletal muscle morphology (Osborn et al., 2017; Wiessner et al., 2017). Moreover, complete loss of INPP5K resulted in embryonic lethality in mice (Ijuin et al., 2008). In agreement with the phenotypes associated with INPP5K loss-of-function, INPP5K is highly expressed in the developing and adult brain, eye, and skeletal muscle (Ijuin et al., 2000). Notably, the overexpression of INPP5K in E17.5 cortical neurons promoted neurite outgrowth and increased the number of processes and branches per neuron (Fink et al., 2017; Kauer et al., 2022).

In this study, we show that INPP5K expression increases during brain development. siRNA-mediated knockdown of INPP5K impaired neuron-like differentiation in N2A cells and interfered with protein glycosylation.

## Methods

All animal experiments were approved by the Thüringer Landesamt für Lebensmittelsicherheit und Verbraucherschutz (TLLV). The experiments were performed on a C57BL/6 background. Mice were housed in a 12-h light/12-h dark cycle and had access to mouse chow *ad libitum*.

### N2A cell differentiation experiments for microscopic analysis

N2A (ATCC) cells were cultured in DMEM GlutaMAX (Sigma-Aldrich) supplemented with 10% [v/v] FBS (Biowest) and 1% [v/v] penicillin/streptomycin (Gibco) at 37°C. The N2A cells were seeded on poly-D-lysine (Thermo Fisher)-coated coverslips. The following day, the cells were treated with differentiation medium [Neurobasal medium 1 g/l glucose (Gibco) + 1X B-27 (Gibco) + 1X sodium pyruvate (Gibco) + 1X glutamine (Gibco) + 1% [v/v] penicillin/streptomycin (Gibco)] and transfected with siRNA against either control (siScr; Dharmacon) or INPP5K (si*Inpp5k*; Thermo Fisher) according to the manufacturer's protocol using Lipofectamine 2000 (Invitrogen). After 4 days of differentiation, the cells were fixed with 100% ice-cold methanol, permeabilized with 0.25% TritonX100, blocked in 5% normal donkey serum (NDS), and stained overnight with primary antibodies at 4°C, followed by incubation with the corresponding secondary antibodies or streptavidin coupled to fluorophores (Invitrogen). The following primary antibodies/lectins were used: biotin WGA (Biozol) 1:50, rabbit anti-INPP5K (Proteintech) 1:100, and mouse anti-PDI (Enzo) 1:300. Nuclei were stained with DAPI (10 μg/ml, Invitrogen). Images were taken with a Keyence microscope (BZ-X800E). Brightfield pictures were used for protrusion analysis, while fluorescence pictures were used for ER and glycosylation analysis. The experiments were performed three times. N2A cell differentiation was morphologically evaluated by measuring the length and numbers of dendrite-like protrusions of first-order, second-order, and third-order protrusions. The length and number of protrusions were measured by tracing protrusions manually with the tool “segmented line” in ImageJ. The length was measured in micrometers with the option “measure.” All single cells from at least five to seven images per condition and experiment were traced manually.

For measuring the total protrusion numbers of individual N2A cells, all traced/visible protrusions per cell were counted for all single cells in at least five to seven images per condition and experiment, and the mean per cell was calculated for each condition.

For measuring the total protrusion length per cell, the length of all traced/visible projections per cell was measured for all single cells from at least five to seven images per condition and experiment, and the mean per cell was calculated for each condition.

To determine the mean protrusion length per order, the length of all traced/visible protrusions per respective protrusion order was measured for each cell and each condition.

### Differentiation of N2A cells upon knockdown of INPP5K

N2A (ATCC) cells were cultured in DMEM GlutaMAX (Sigma-Aldrich) supplemented with 10% [v/v] FBS (Biowest) and 1% [v/v] penicillin/streptomycin (Gibco) at 37°C. The N2A cells were seeded in 10-cm cell culture dishes (Greiner). The following day, the cells were treated with differentiation medium [Neurobasal medium 1 g/L glucose (Gibco) + 1X B-27 (Gibco) + 1X sodium pyruvate (Gibco) + 1X glutamine (Gibco) + 1% [v/v] penicillin/streptomycin (Gibco)] and at the same time transfected with siRNA against either control (siScr; Dharmacon) or INPP5K (si*Inpp5k*; Thermo Fisher) according to the manufacturer's protocol using Lipofectamine 2000 (Invitrogen). After 3 days of differentiation, the cells were harvested and lysed in RIPA buffer [50 mM Tris-HCl pH 7.4, 150 mM NaCl, 1% [v/v] NP-40, 1% [w/v] sodium deoxycholate, 0.1% [w/v] SDS, 1 mM EDTA] and a complete protease inhibitor (Roche). Cell homogenates were centrifuged at 10,000 *g*, and the protein concentration in the supernatant was determined using the BCA assay kit (Thermo Fisher). Samples were stored at −20°C until further use. The experiments were performed three times.

### Knockdown of INPP5K in differentiated N2A cells

N2A (ATCC) cells were cultured in DMEM GlutaMAX (Sigma-Aldrich) supplemented with 10% [v/v] FBS (Biowest), 1% [v/v] penicillin/streptomycin (Gibco) at 37°C. The N2A cells were seeded on poly-D-lysine (Thermo Fisher) coated coverslips. The following day, the cells were treated with differentiation medium [Neurobasal medium 1 g/L glucose (Gibco) + 1X B-27 (Gibco) + 1X sodium pyruvate (Gibco) + 1X glutamine (Gibco) + 1% [v/v] penicillin/streptomycin (Gibco)] and allowed to differentiate for 3 days. Then, the cells were transfected with siRNA against either control (siScr; Dharmacon) or INPP5K (si*Inpp5k*; Thermo Fisher) according to the manufacturer's protocol using Lipofectamine 2000 (Invitrogen). After 4 days of differentiation, cells were fixed and processed as described above. The experiments were performed three times.

### Protein isolation from the brain

Pregnant mice or pups were sacrificed, and the brain tissues were dissected. The brain tissue was obtained from 3 E11.5 and 3 E18.5 embryos as well as from 3 postnatal day (P) 3 and 3 P18 pups. Tissue lysates were prepared as previously described (Franzka et al., 2022). Briefly, samples were homogenized with the Potter S tissue homogenizer (Sartorius) in RIPA buffer [50 mM Tris-HCl pH 7.4, 150 mM NaCl, 1% [v/v] NP-40, 1% [w/v] sodium deoxycholate, 0.1% [w/v] SDS, 1 mM EDTA] and a complete protease inhibitor (Roche). After sonication, homogenates were spun down at 16,900 *g* to remove nuclei and insoluble debris. Protein concentration in the supernatant was determined using the BCA assay kit (Thermo Fisher) and then stored at −80°C until further use.

### Western blot

Proteins isolated from three embryos/pups at the indicated developmental stages or from N2A cells (transfected with siRNA against either control or INPP5K) from three individual experiments were denatured at 90°C for 5 min in Laemmli buffer (4X Laemmli buffer: 50% glycerol, 5% SDS, 0.25% 1.5 M Tris pH 6.8, 30% β-mercaptoethanol, and 0.001% bromophenol blue, ddH_2_O). After separation by SDS-PAGE, proteins were transferred onto PVDF membranes (Whatman). Membranes were blocked in 2% BSA and incubated with primary antibodies at appropriate dilutions overnight at 4°C. The following primary antibodies or lectins were used: rabbit anti-INPPK (Proteintech) 1:500, rabbit anti-GAPDH (Proteintech) 1:1,000, biotin WGA (Biozol) 1:300, biotin PNA (Bioworld) 1:300, biotin SNL (Biozol) 1:300, biotin RCAI (Biozol) 1:300, biotin Con A (Biozol) 1:300, biotin LCH (Ey Laboratories) 1:300, and biotin MAL (Biomol) 1:300. Primary antibodies or lectins were detected with HRP-conjugated secondary antibodies or HRP-conjugated streptavidin. Detection was performed using the Clarity Western ECL Substrate Kit (BioRad). The quantification of bands was performed using ImageJ.

Coomassie blue staining of PVDF membranes was performed as described previously (Franzka et al., 2021). For Coomassie blue staining of transferred proteins, PVDF membranes were fixed for 3 min (10% acetic acid, 40% EtOH), stained in Coomassie blue solution (0.1% Brilliant Blue R (Serva), 45% EtOH, 10% acetic acid) for 5 min, destained (10% acetic acid, 20–40% EtOH), rinsed in H_2_O, and imaged.

### Immunofluorescence analysis of embryo sections

Pregnant mice or pups were sacrificed, and whole embryos or brain tissues were dissected. Tissue was obtained from each of the three E11.5, E13.5, and E18.5 embryos, as well as from the three postnatal day (P) 3 and P18 pups. The tissues were frozen in TissueTek (company) on dry ice and cryo-sectioned. Sections were dried, fixed in 4% paraformaldehyde (PFA), permeabilized with 0.25% TritonX100, blocked in 5% normal goat serum (NGS), and stained overnight at 4°C with primary antibodies or lectins, followed by incubation with the corresponding secondary antibodies or streptavidin coupled to fluorophores (Invitrogen). The following primary antibodies or lectins were used: biotin WGA (Biozol) 1:100, rabbit anti-INPP5K (Proteintech) 1:100, and biotin PNA (Bioworld) 1:100. Nuclei were stained with DAPI (10 μg/ml, Invitrogen). Images were taken using a Keyence microscope BZ-X800E.

### Statistical analysis

For statistical analysis, raw data were analyzed for normal distribution with the Kolmogorov–Smirnov test or by graphical analysis using the Box-Plot and QQ-Plot in GraphPad Prism 9. If appropriate, we either used a one-way ANOVA, a two-way ANOVA, or a two-tailed Student's *t*-test. ^*^p < 0.05, ^**^p < 0.01, ^***^p < 0.001, ^****^p < 0.0001, and ns, not significant. For statistical analysis, we used GraphPad Prism 9. For all data, means with the standard error of the mean (SEM) and individual data points with the SEM are shown.

## Results

### INPP5K expression increases in the developing mouse brain

**Figure 1:**
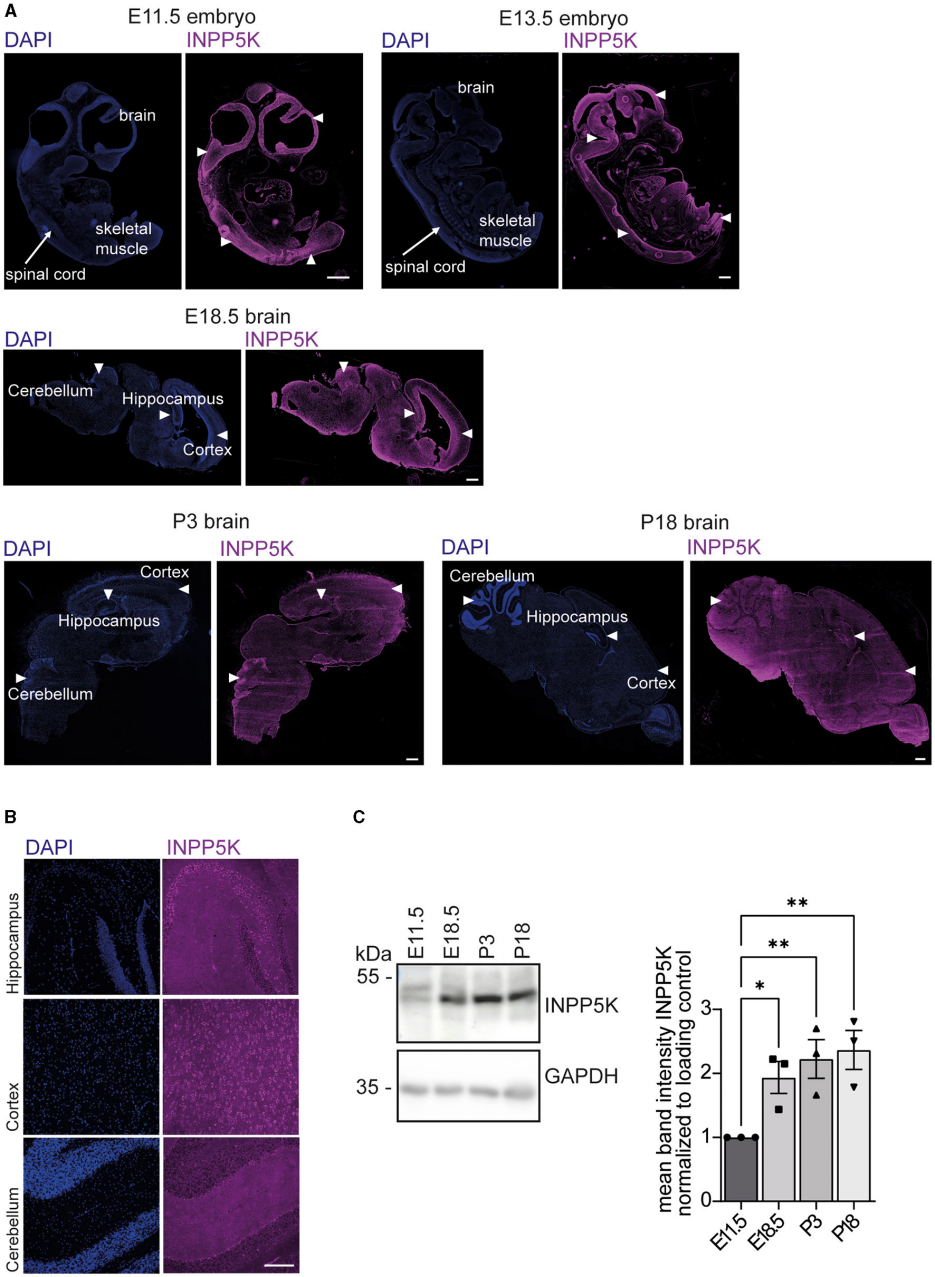
INPP5K expression increases in the developing mouse brain. **(A)** Immunofluorescence of sagittal whole embryo and brain sections at indicated time points stained for INPP5K (scale bar: 500 μm). Nuclei were labeled with DAPI. White arrowheads indicate prominent labeling of INPP5K in whole embryo sections. In E18.5 and P3 brain sections, arrowheads mark the hippocampus, the cortex, and the cerebellum. **(B)** Immunolabeling of INPP5K expression in a magnification of the hippocampus, cortex, and cerebellum of sagittal brain sections from P18 mice (scale bar: 100 μm). **(C)** Immunoblot analysis of brain tissue lysates at the indicated time points showed a strong increase in INPP5K abundance during brain development. GAPDH served as a loading control (*n* = 3 samples per time point, one-way ANOVA with Fisher's LSD test). Quantitative data are presented as mean ± SEM with individual data points. *p < 0.05, **p < 0.01.

To analyze the expression pattern of INPP5K during mouse development, we stained sagittal whole embryo or brain sections of different developmental stages with an antibody directed against INPP5K (Figure 1A, Supplementary Figures 2, 3). Prominent INPP5K signals were found in developing skeletal muscles and in the central nervous system (Figure 1A, Supplementary Figures 2, 3). In the brain, INPP5K expression was detected in various regions, especially in the hippocampus, the cortex, and Purkinje cells (Figure 1B). Excluding the unspecific binding of secondary antibodies, no signals were observed when the primary antibody was omitted (Supplementary Figure 4).

We next assessed the abundance of INPP5K in protein lysates from mouse brains isolated at different time points of embryonic development. Notably, INPP5K levels increased dramatically during embryonic as well as early postnatal (P) brain development (Figure 1C, Supplementary Figure 5).

Taken together, these findings suggest a pivotal role for INPP5K in the brain.

### INPP5K knockdown impairs the differentiation of N2A cells upon serum starvation

**Figure 2:**
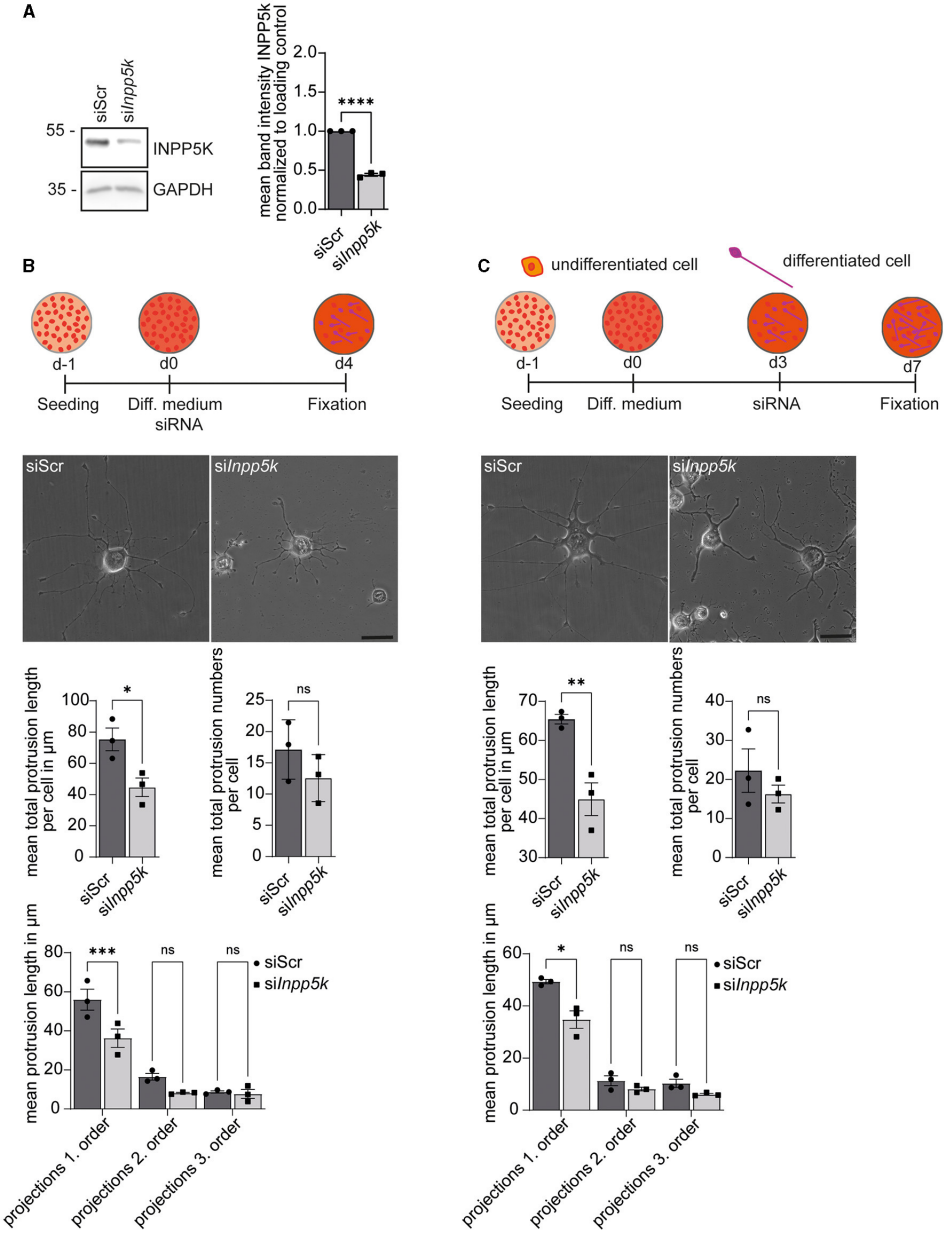
INPP5K knockdown impairs the differentiation of N2A cells upon serum starvation. **(A)** Immunoblot analyses confirmed the efficient knockdown of INPP5K. GAPDH served as a loading control. **(B)** Experimental design and representative images of N2A cells (scale bar: 50 μm) and the quantification of the length and numbers of protrusions [*n* = 3 experiments with 5–7 pictures per condition and experiment with 5–15 cells per picture (35–75 cells per experiment)], Student's *t*-test or one-way ANOVA with Fisher's LSD test. **(C)** Experimental design and representative images of N2A cells (scale bar: 50 μm) and quantification of the length and number of protrusions [*n* = 3 experiments with 5–7 pictures per condition and experiment with 5–15 cells per picture (35–75 cells per experiment)], Student's *t*-test or one-way ANOVA with Fisher's LSD test. Quantitative data are presented as mean ± SEM with individual data points. *p < 0.05, **p < 0.01, ***p < 0.001, ****p < 0.0001; ns, not significant.

To assess whether INPP5K is relevant for cell differentiation, we transfected N2A cells with siRNAs directed against INPP5K (si*Inpp5k*) or a scrambled control siRNA (siScr; Figure 2A, Supplementary Figure 5). We then serum-deprived the transfected N2A cells to induce neuronal-like differentiation and measured the numbers and length of first-, second-, and third-order protrusions 4 days later. While the number of protrusions did not differ from controls, the length of first-order dendrites was significantly decreased after the siRNA-mediated knockdown of INPP5K (Figure 2B, Supplementary Figure 1A). Dendrite numbers did not differ between conditions (Figure 2B, Supplementary Figure 1A).

To assess whether INPP5K is required for the maintenance of differentiated N2A cells, we induced differentiation for 4 days before we knocked down INPP5K. The number and length of the protrusions were evaluated 4 days later. While control cells maintained long primary protrusions, the length of primary protrusions was significantly reduced after siRNA-mediated knockdown of INPP5K in differentiated N2A cells (Figure 2C, Supplementary Figure 1B).

### Knockdown of INPP5K in N2A cells interferes with protein glycosylation

**Figure 3:**
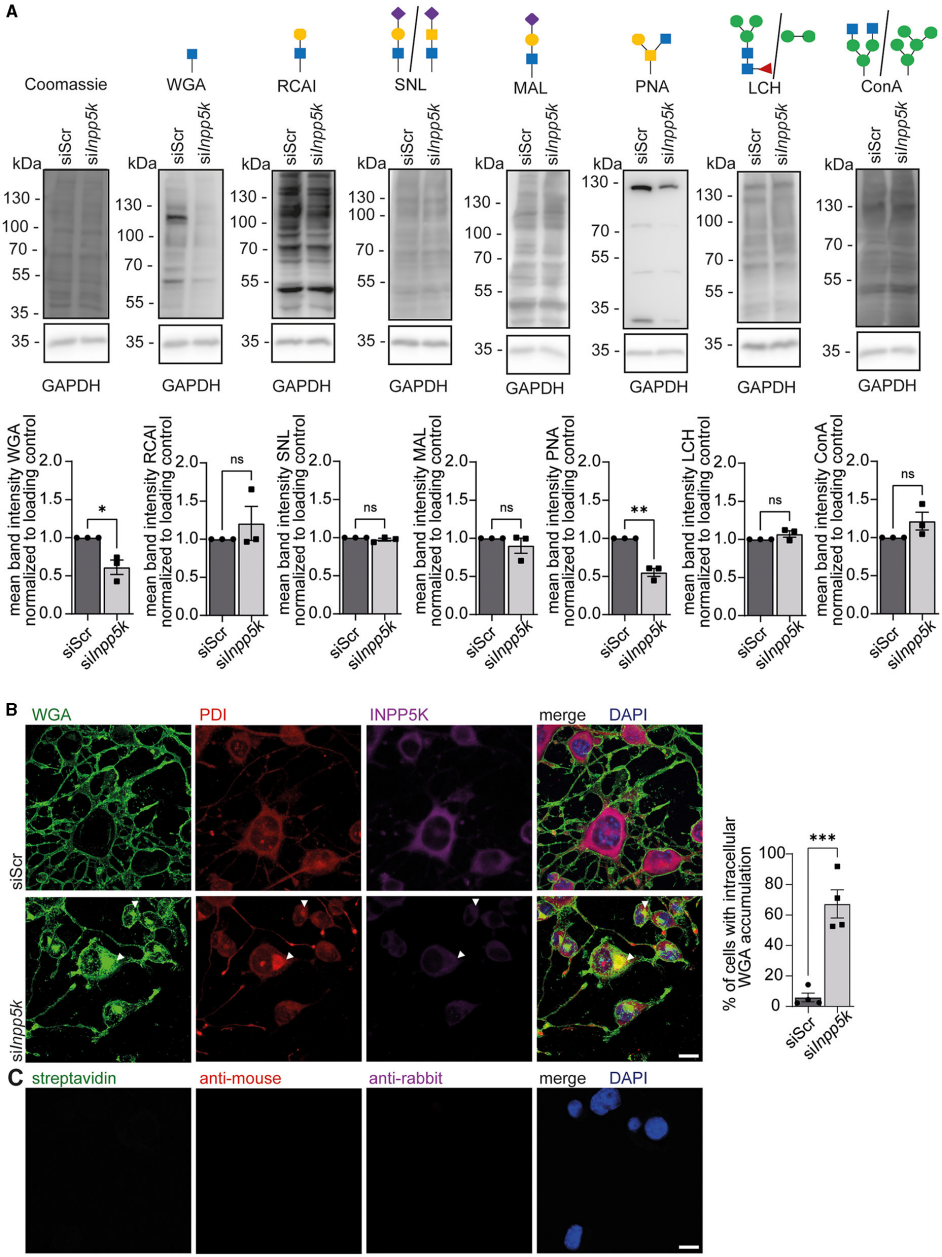
Knockdown of INPP5K in N2A cells interferes with protein glycosylation. **(A)** Representative Coomassie stained membranes and membranes probed with different biotinylated lectins, i.e., WGA, RCAI, SNL, MAL, PNA, LCH, and Con A, and the respective quantifications. GAPDH and Coomassie staining served as a loading control. Green circle = mannose, yellow circle = galactose, blue square = *N*-acetylglucosamine, yellow square = *N*-acetylgalactosamine, violet diamond = N-glycolylneuraminic acid, and red triangle = fucose. Lectin signals were normalized to GAPDH. N2A cells were transfected with either scRNA or siRNA to knock down INPP5K, harvested, and the respective protein lysates separated by SDS-PAGE and transferred to membranes (*n* = 3 experiments, Student's *t*-test). **(B)** Staining of N2A cells transfected with either scRNA or siRNA to knock down INPP5K with WGA, anti-PDI, anti-INPP5K, and DAPI to label nuclei (scale bar 10 μm, *n* = 4 experiments with 200–5,000 cells per condition and experiment, Student's *t*-test). White arrowheads indicate examples of cells with WGA-positive deposits co-labeling with the ER marker PDI. Cells with intracellular deposits labeled by WGA were quantified. **(C)** As a control, we also incubated N2A cells with either secondary antibodies alone or fluorophore-coupled streptavidin, which was used to detect biotinylated WGA (scale bar 10 μm). Quantitative data are presented as mean ± SEM with individual data points. *p < 0.05, **p < 0.01, ***p < 0.001; ns, not significant.

Patients with MDCCAID suffer from congenital muscular dystrophy with hypoglycosylation of dystroglycan (Osborn et al., 2017; Wiessner et al., 2017). To study whether the knockdown of INPP5K in N2A cells alters protein glycosylation, we isolated protein lysates of differentiated N2A cells transfected with either scRNA or siRNAs to knock down INPP5K upon the induction of differentiation. After separation by SDS-PAGE and blotting, membranes were incubated with different lectins to detect specific glycan structures. Upon knockdown of INPP5K, signals were diminished for wheat germ agglutinin (WGA), which detects *N*-acetylglucosamine residues, and for peanut agglutinin (PNA), which recognizes non-sialylated β(1–3)-linked galactose on *N*-acetylgalactosamine residues (Figure 3A, Supplementary Figure 5). No differences were found in non-sialylated/sialylated galactose/glucosamine on *N*-acetylglucosamine residues, as detected by ricinus communis agglutinin I (RCAI), sambucus nigra lectin (SNL) or maackia amurensis lectin (MAL). Similarly, no differences were found in fucosylated/mannose-carrying residues, as detected by the lens culinaris lectin (LCH) or concanavalin A (Con A; Figure 3A, Supplementary Figure 5).

We then fixed N2A cells either transfected with the scRNA or the siRNAs directed against INPP5K at the same time as the induction of differentiation and co-stained for *N*-acetylglucosamine residues and the luminal ER protein phosphodiesterase (PDI; Figures 3B, C). Consistent with our immunoblots, INPP5K signals were diminished upon knockdown of INPP5K. Notably, cells showing reduced INPP5K signals showed a prominent intracellular accumulation of WGA and a co-localization of PDI with WGA signals, suggesting that glycoproteins were retained within the ER (Figure 3B).

In summary, the knockdown of INPP5K in N2A cells affects protein glycosylation.

### WGA and PNA reactivities increase during mouse brain development

Because WGA and PNA reactivities were diminished in protein lysates of cells transfected with siRNAs to knock down INPP5K (Figure 3), we wondered about the developmental profile for *N*-acetylglucosamine residues detected by WGA and non-sialylated β(1–3)-linked galactose on *N*-acetylgalactosamine residues detected by peanut agglutinin (PNA).

**Figure 4:**
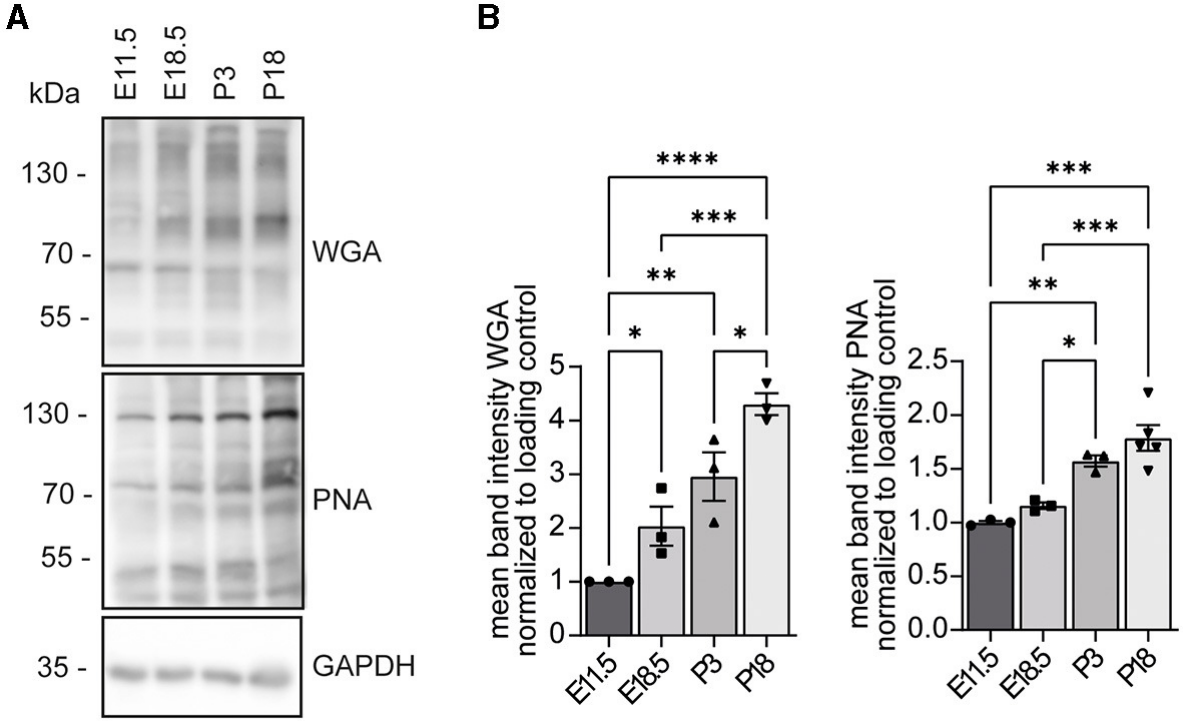
WGA and PNA reactivities increase during mouse brain development. **(A)** Protein lysates of mouse brains at the indicated developmental stages were separated by SDS-PAGE, blotted, and probed with either WGA or PNA. GAPDH served as a loading control. **(B)** Analysis of WGA and PNA signals shown in **(A)** (*n* = 3 samples per time point, one-way ANOVA with Fisher's LSD test). Quantitative data are presented as mean ± SEM with individual data points. *p < 0.05, **p < 0.01, ***p < 0.001, ****p < 0.0001.

We assessed the abundance of *N*-acetylglucosamine residues and non-sialylated β(1–3)-linked galactose on *N*-acetylgalactosamine residues in mouse brains isolated at different time points of embryonic and postnatal development. The respective protein lysates were separated by SDS-PAGE and blotted. For both glycan structures, we observed a strong increase between E11.5 and P18 (Figure 4, Supplementary Figures 2–5).

## Discussion

Mutations that decrease the enzymatic activity of INPP5K are associated with an autosomal recessive human disorder characterized by congenital muscular dystrophy with hypoglycosylation of dystroglycan in combination with cataracts, intellectual disability, and short stature (Osborn et al., 2017; Wiessner et al., 2017), while some polymorphisms in INPP5K have been associated with Parkinson's disease (Zhu et al., 2018). Remarkably, the total knockout caused embryonic lethality in mice (Ijuin et al., 2008). In agreement with the phenotype of patients and confirming previous reports (Ijuin et al., 2000), INPP5K is strongly expressed in the developing brain and skeletal muscles of the mouse and further increases with embryonic and postnatal brain development. Suggesting a major role in neuronal differentiation, the overexpression of INPP5K in E17.5 cortical neurons promoted neurite outgrowth and increased the number of processes and branches per neuron (Fink et al., 2017; Kauer et al., 2022). Here, we assessed the consequences of its siRNA-mediated knockdown in neuroblastoma-derived N2A cells. Under serum starvation, N2A cells can be differentiated into cells with complex protrusions resembling dendrites (Evangelopoulos et al., 2005). Remarkably, the knockdown of INPP5K impaired the outgrowth and the maintenance of dendrite-like protrusions in N2A cells, which may relate to the observation that patients with INPP5K loss-of-function show intellectual disability and brain abnormalities, especially cerebellar or global brain atrophy (Osborn et al., 2017; Wiessner et al., 2017; D'Amico et al., 2020; Hathazi et al., 2021).

The subcellular localization of INPP5K can vary depending on the specific cellular requirements. While a large pool localizes to the ER, INPP5K can be recruited to the plasma membrane to downregulate PI(3,4,5)P_3_ signaling upon growth factor stimulation (Gurung et al., 2003). In *Caenorhabditis elegans*, INPP5K together with atlastin-1 was shown to be involved in maintaining the non-uniform, somatodendritic enrichment of neuronal ER/plasma membrane contacts in their soma and dendrites, which are mostly absent in axons (Loncke et al., 2023). The hypoglycosylation of dystroglycan in patients with INPP5K loss-of-function further connects INPP5K with ER and Golgi functions. In agreement, the knockdown of INPP5K in N2A cells clearly affected the glycosylation of proteins, as evidenced by reduced signal intensities for *N*-acetylglucosamine residues or non-sialylated β(1–3)–linked galactose. Because *N*-acetylglucosamine is the first sugar residue attached to the amide group for *N*-glycosylation of proteins (Varki et al., 2009; Breloy and Hanisch, 2018), loss of INPP5K can have important consequences for glycoproteins, such as α-dystroglycan or PDI, carrying *N*-glycosylated sugar residues. Aberrant protein glycosylation can interfere with proper protein folding and may thus explain the accumulation of glycoproteins carrying *N*-acetylglucosamine within the ER upon knockdown of INPP5K.

## Data availability statement

The raw data supporting the conclusions of this article will be made available by the authors, without undue reservation.

## Ethics statement

The animal study was reviewed and approved by the Thüringer Landesamt für Lebensmittelsicherheit und Verbraucherschutz (TLLV). The study was conducted in accordance with the local legislation and institutional requirements.

## Author contributions

AM: Writing – original draft, Formal analysis. LG: Writing – original draft, Methodology. PF: Writing – review & editing, Writing – original draft, Supervision, Project administration, Methodology, Investigation, Funding acquisition, Conceptualization. CH: Writing – original draft, Writing – review & editing, Funding acquisition.
