# Supplementary material for: Knockdown of INPP5K compromises the differentiation of N2A cells
Source: Front Mol Neurosci. 2024 Mar 15;17:1356343. doi: 10.3389/fnmol.2024.1356343 (PMC10979461; doi:10.3389/fnmol.2024.1356343)
Supplement: Supplementary file 1 [file Data_Sheet_1.docx]

Supplementary Material

**Knockdown of INPP5K compromises the differentiation of N2A cells**

The following supporting information is available: Supplementary Figure 1. INPP5K knockdown impairs the differentiation of N2A cells. Supplementary Figure 2. INPP5K and non-sialylated galactose staining patterns during mouse development. Supplementary Figure 3. INPP5K and *N*-acetylglucosamine staining patterns during mouse development. Supplementary Figure 4. INPP5K expression during mouse development. Supplementary Figure 5. Full Western blots shown in main figures.


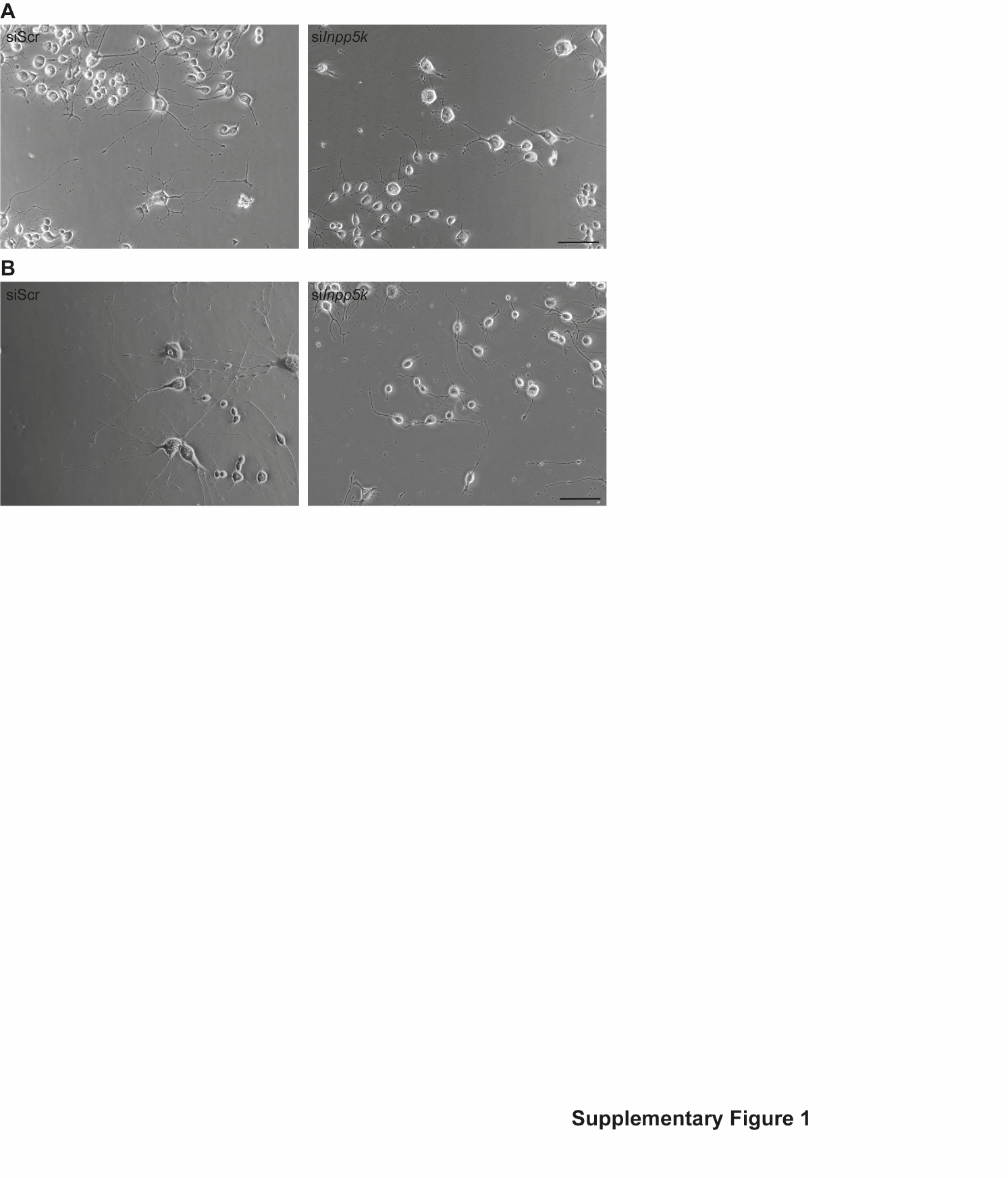


**Supplementary Figure 1. INPP5K knockdown impairs the differentiation of N2A cells. A)** Representative images of N2A cells (scale bar: 100 µm) for Figure 2B taken with a 20X objective (whole image). **B)** Representative images of N2A cells (scale bar: 100 µm) for Figure 2C taken with a 20X objective (whole image).

**
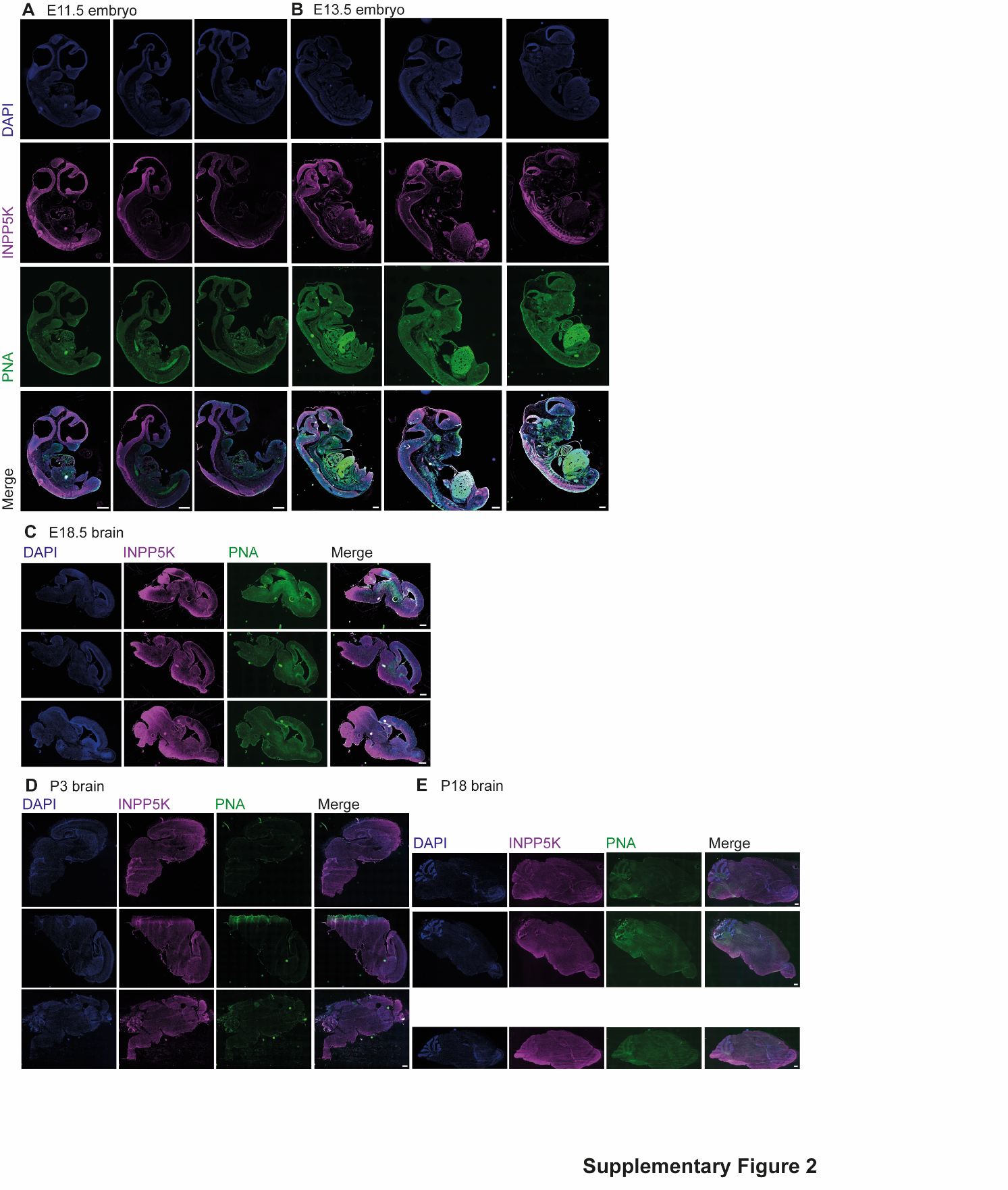
**

**Supplementary Figure 2. INPP5K and non-sialylated galactose staining patterns during mouse development.** Immunofluorescence analysis of the expression of INPP5K and PNA signals of sagittal whole embryo and brain sections at indicated time-points (scale bar: 500 µm). Nuclei were labeled with DAPI. For each time-point, three mice were assessed. Images are shown for **A)** E11.5 sagittal whole embryo sections, **B)** E13.5 sagittal whole embryo sections, **C)** E18.5 sagittal brain sections, **D)** P3 sagittal brain sections and **E)** P18 sagittal brain sections.

**
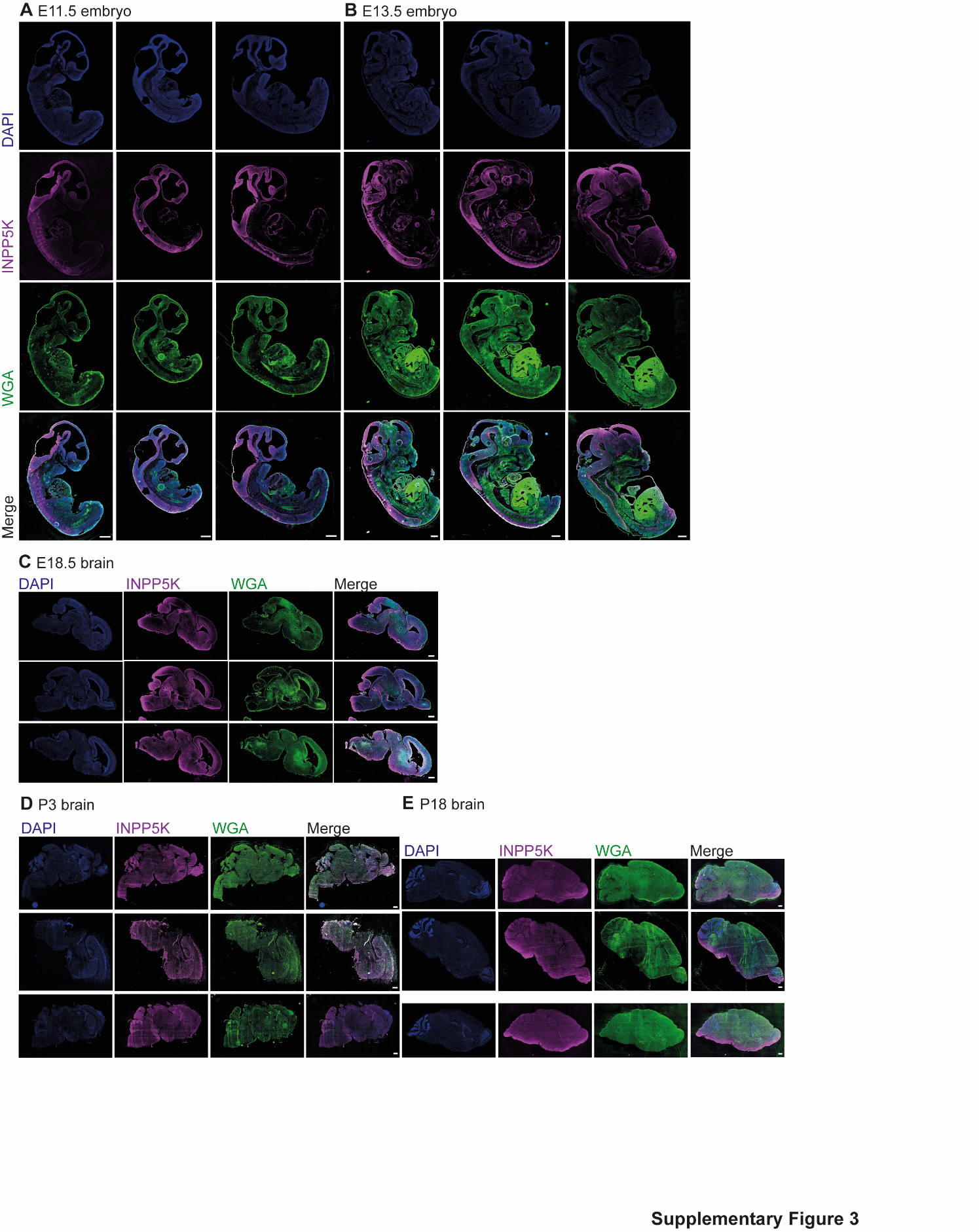
**

**Supplementary Figure 3. INPP5K and detect *N*-acetylglucosamine staining patterns during mouse development.** Immunofluorescence analysis of the expression of INPP5K and WGA signals of sagittal whole embryo and brain sections at indicated time-points (scale bar: 500 µm). Nuclei were labeled with DAPI. For each time-point, three mice were assessed. Images are shown for **A)** E11.5 sagittal whole embryo sections, **B)** E13.5 sagittal whole embryo sections, **C)** E18.5 sagittal brain sections, **D)** P3 sagittal brain sections and **E)** P18 sagittal brain sections.

**
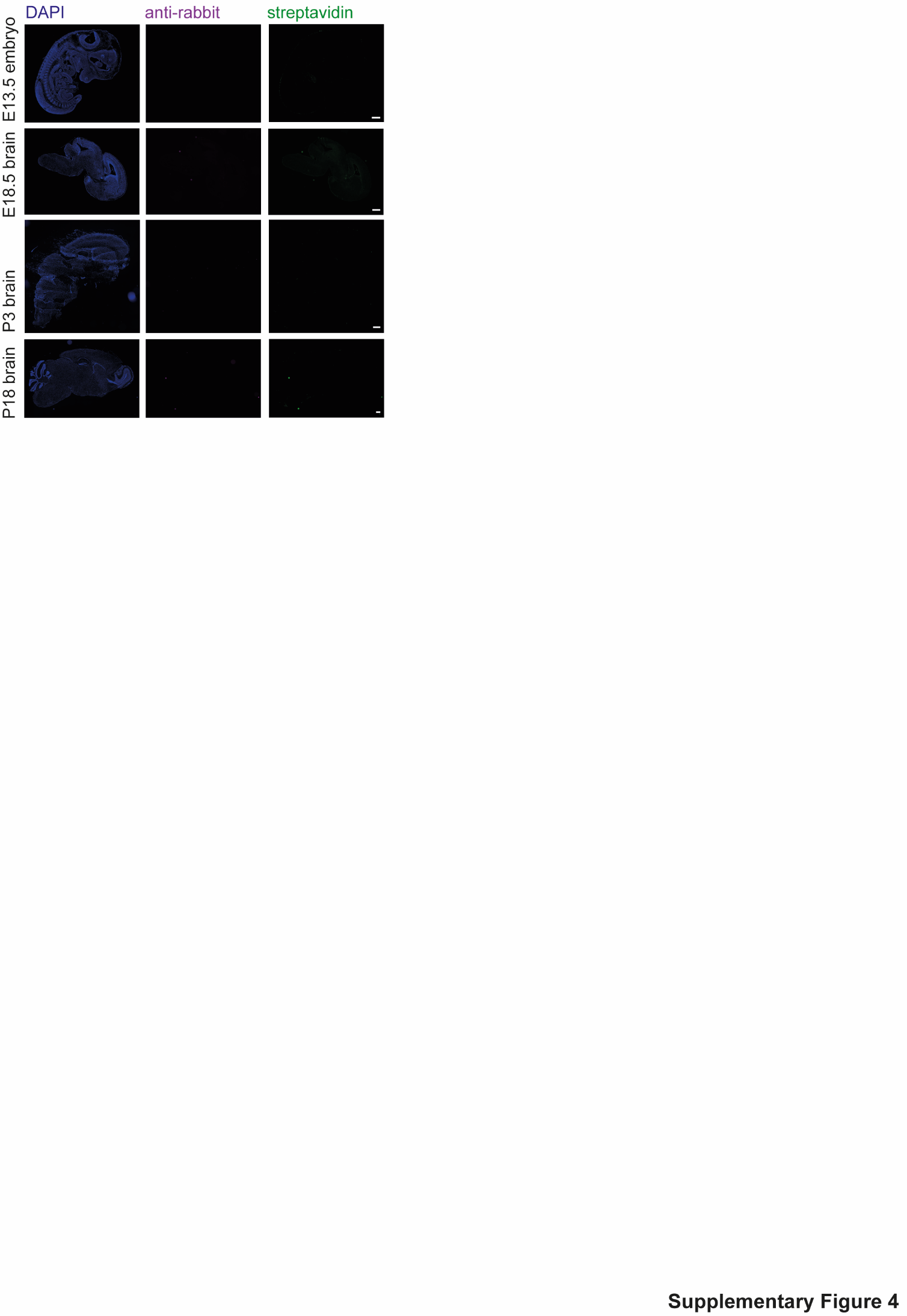
**

**Supplementary Figure 4. INPP5K expression during mouse development.** Control immunofluorescence staining with the secondary anti-rabbit-coupled 555 Alexa Fluorophore antibody and the streptavidin-coupled 488 Alexa Fluorophore alone, which was used to detect WGA and PNA (scale bar: 500 µm).

**Supplementary Figure 5. Full Western blots shown in main figures.**
