# Supplementary material for: Knockdown of INPP5K compromises the differentiation of N2A cells
Source: Front Mol Neurosci. 2024 Mar 15;17:1356343. doi: 10.3389/fnmol.2024.1356343 (PMC10979461; doi:10.3389/fnmol.2024.1356343)

Full Western Blots for Figure 1C

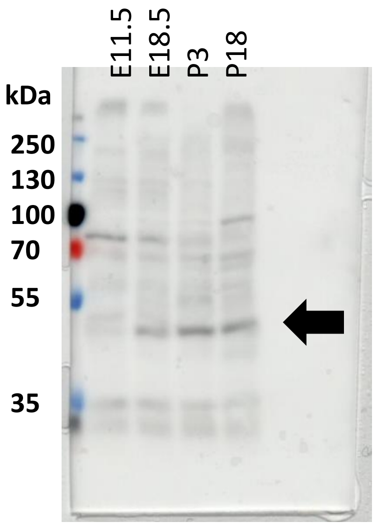

Rabbit anti-INPP5K
Lower exposotion time

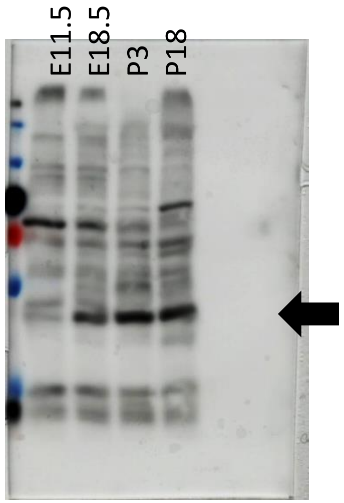

Rabbit anti-INPP5K
Higher exposition time

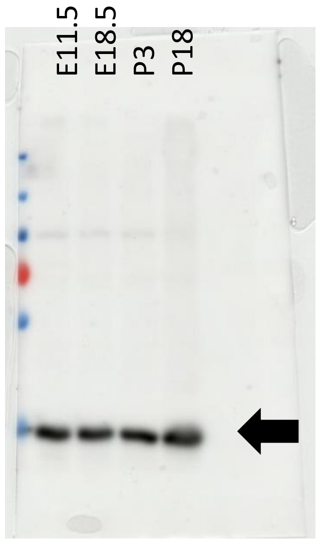

Rabbit anti-GAPDH

Full Western Blots for Figure 2A

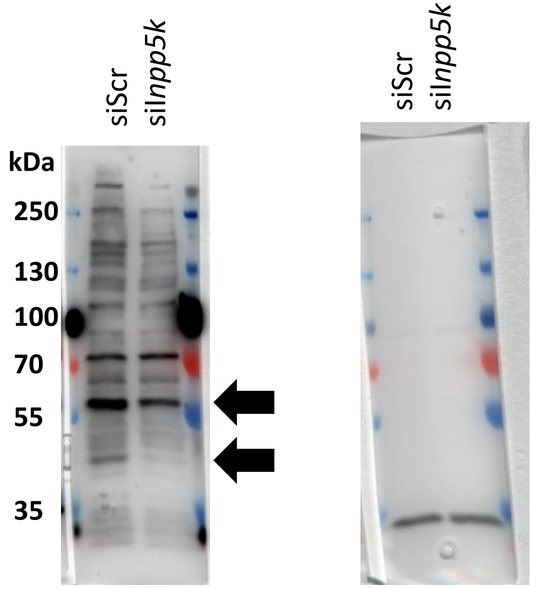

Rabbit anti-INPP5K

Rabbit anti-GAPDH

Full Western Blots for Figure 3A (all lectins are biotinylated)

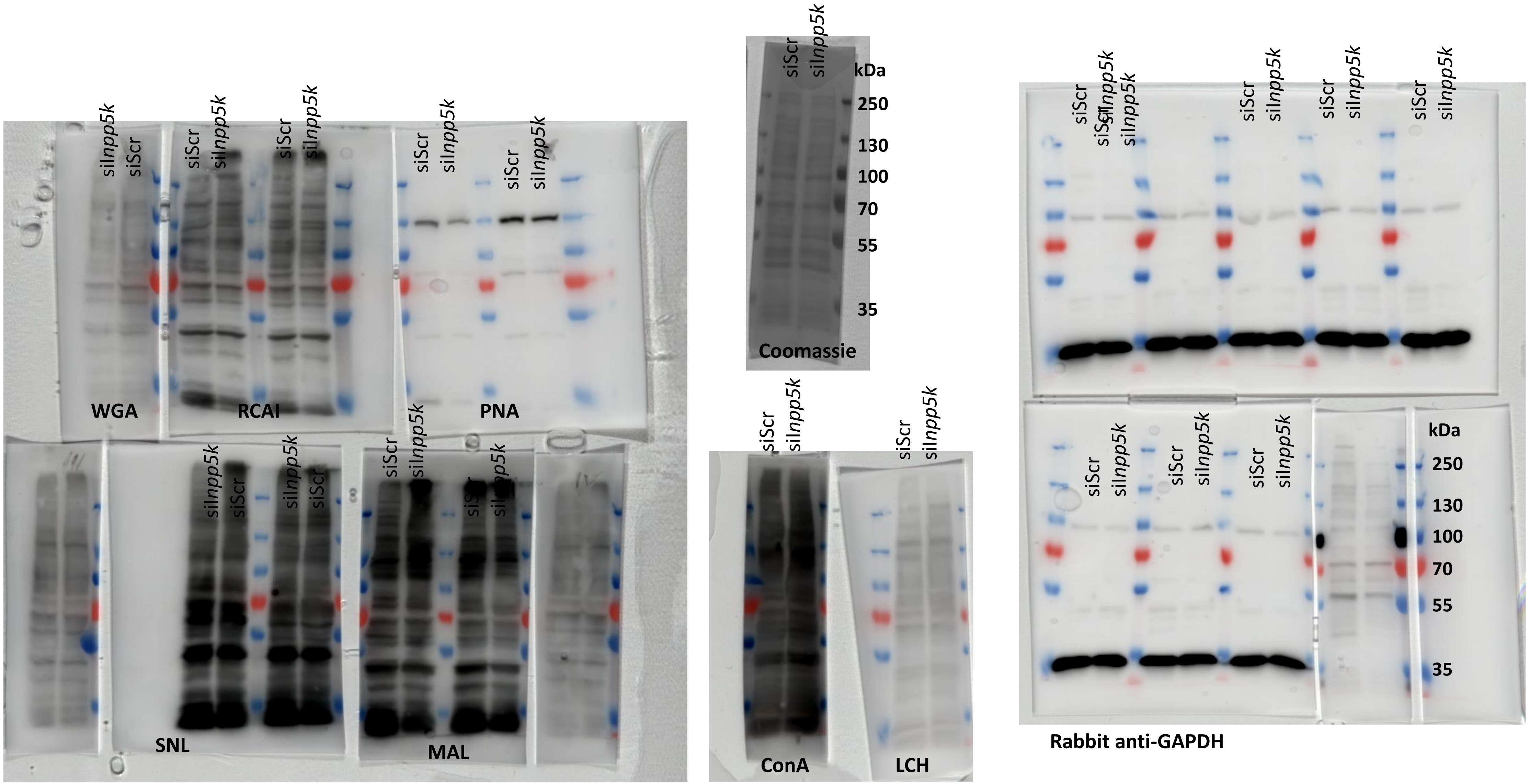

Full Western Blots for Figure 4A (all lectins are biotinylated)

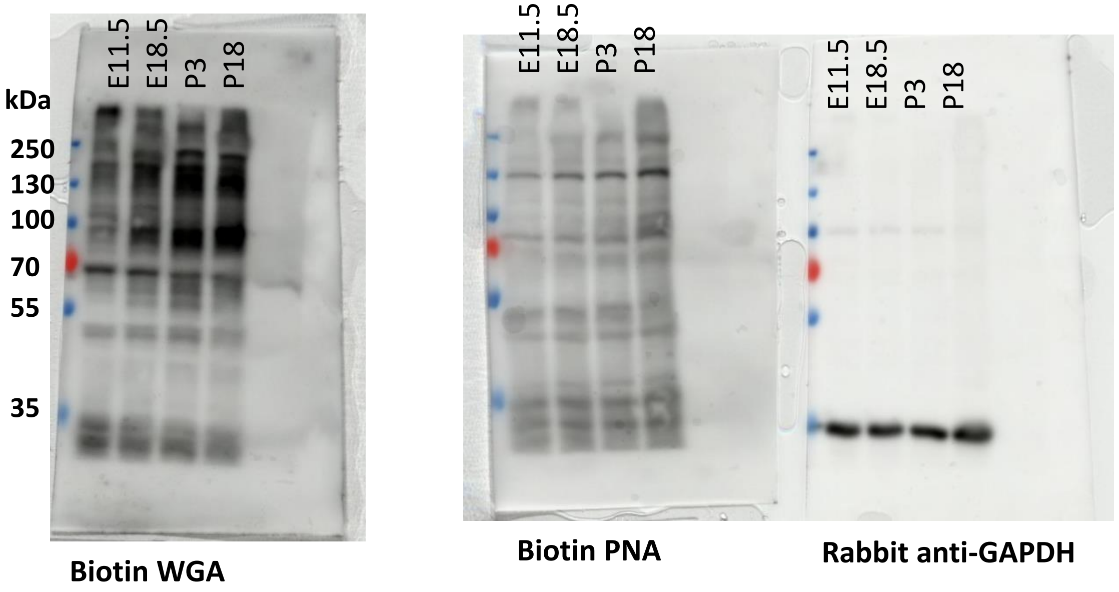

Supplement: Supplementary file 2 [file Presentation_1.pdf]
